# Passage and reversal effects on gene expression of bovine meniscal fibrochondrocytes

**DOI:** 10.1186/ar2293

**Published:** 2007-09-13

**Authors:** Najmuddin J Gunja, Kyriacos A Athanasiou

**Affiliations:** 1 Department of Bioengineering, Rice University, PO Box 1892, Houston, TX 77251, USA

## Abstract

The knee meniscus contains a mixed population of cells that exhibit fibroblastic as well as chondrocytic characteristics. Tissue engineering studies and future therapies for the meniscus require a large population of cells that are seeded on scaffolds. To achieve this, monolayer expansion is often used as a technique to increase cell number. However, the phenotype of these cells may be significantly different from that of the primary population. The objective of this study was to investigate changes in meniscal fibrochondrocytes at the gene expression level over four passages using quantitative real-time reverse transcriptase polymerase chain reaction. Cells from the inner two-thirds of bovine medial menisci were used. Four extracellular matrix (ECM) molecules, commonly found in the meniscus, were investigated, namely collagen I, collagen II, aggrecan and cartilage oligomeric matrix protein (COMP). In addition, primary and passaged meniscus fibrochondrocytes were placed on surfaces coated with collagen I or aggrecan protein to investigate whether any gene expression changes resulting from passage could be reversed. Collagen I expression was found to increase with the number of passages, whereas collagen II and COMP expression decreased. Collagen I and aggrecan surface coatings were shown to downregulate and upregulate collagen I and COMP expression levels, respectively, in passaged cells. However, decreases in collagen II expression could not be reversed by either protein coating. These results indicate that although monolayer expansion results in significant changes in gene expression in meniscal fibrochondrocytes, protein coatings may be used to regain the primary cell expression of several ECM molecules.

## Introduction

The meniscus is a wedge-shaped fibrocartilaginous tissue located in the knee joint. As reviewed elsewhere, it serves several mechanical functions including shock absorption, load transmission, joint stability and joint lubrication [1,2]. Injuries to the meniscus can result in significant pain and discomfort to the patient, as well as in increasing the average stress in the knee joint, causing damage to the articular cartilage on the femoral and tibial surfaces [3]. The ability of the meniscus to heal intrinsically is limited to the vascular regions of the tissue. Thus, tissue engineering is a promising treatment modality to replace avascular sections of the meniscus [2].

The term fibrochondrocyte or fibrocartilage cell has often been used to describe the cells of the meniscus [4-7]. However, recent characterization studies have led to the identification of different cell populations within the tissue [2,8]. McDevitt and colleagues [8] divided the meniscal cell population into three distinct groups: fibrochondrocytes, fibroblast-like cells, and cells of the superficial zone. Fibrochondrocytes, as defined by the authors, are cells that are localized in the middle and inner meniscus and express both collagen I and collagen II. They can be identified by their round or oval shape and by the presence of a pericellular matrix. The extracellular matrix (ECM) in this region consists mainly of collagens I and II, in a 2:3 ratio, which are responsible for providing structural and tensile properties to the tissue [9,10]. Fibroblast-like cells are found mainly in the outer one-third of the tissue and lack a pericellular matrix. The ECM in this region is predominantly collagen I [2,11]. Cells of the superficial zone are located below the surface of the tissue and can be identified by their fusiform shape and lack cytoplasmic projections. In this experiment, we use cells from the inner two-thirds of the meniscus; thus, most of the cells present are fibrochondrocytes. In addition to the presence of collagen I and II in the inner two-thirds of the meniscus, several other proteoglycans and glycoproteins can also be found. The major meniscal proteoglycan is aggrecan and its main function is to provide compressive properties of the meniscus, especially to the inner one-third, which is predominantly under compressive load [12]. Cartilage oligomeric matrix protein (COMP), a pentameric glycoprotein that influences collagen fibril formation, can also be identified in the inner two-thirds of the meniscus [13]. Also present, in smaller quantities, are small leucine-rich proteoglycans, biglycan and decorin, that interact with growth factors as well as influence fibrillogenesis [7].

Current cellular approaches for meniscus tissue engineering usually involve autologous meniscus cells [14,15]. However, there are too few primary cells in any one animal to be seeded on a scaffold. To overcome this, an approach often employed is to expand autologous cells in monolayer until the cell number is sufficient for the study. A caveat with this technique is that primary cells may dedifferentiate *in vitro *in monolayer culture. This has been shown consistently with articular cartilage [16,17]. Gene expression studies with primary chondrocytes show that they express predominantly collagen II. However, after one passage, the collagen II expression decreases and the cells begin to express collagen I, which is indicative of a fibroblastic phenotype [18,19]. In an effort to reverse lost gene expression in articular cartilage and temporomandibular joint (TMJ) disc fibrochondrocytes, several growth factors, surface protein coatings and three-dimensional scaffolds have been investigated [18,20-22]. However, corresponding passage and gene expression reversal studies for the meniscus are absent. Hence, understanding the state of expanded meniscal fibrochondrocytes before embarking on long-term tissue engineering studies may be prudent.

The goal of this experiment was, thus, twofold. The first was to determine the effects of passage on the gene expression of important ECM molecules (collagen I, collagen II, aggrecan and COMP) produced by meniscal fibrochondrocytes. The hypothesis was that, much like articular chondrocytes, meniscal fibrochondrocytes would exhibit phenotypic changes in monolayer culture. The second was to reverse any changes in gene expression incurred during passage by plating passaged meniscus cells on either an aggrecan or a collagen I protein coating.

## Materials and methods

### Cell harvesting

Medial menisci were isolated from six 1-week-old calf knees (Research 87 Inc., Boston, MA, USA)) by exposing the knee joint under aseptic conditions using scalpel blades. The procedures used were in strict accordance with the National Institutes of Health Guidelines on the Care and Use of Laboratory Animals. Ethics approval was obtained from Rice University before commencement of the study.

Each meniscus was taken to a cell culture hood, washed with autoclaved PBS and transferred to a solution containing 2% penicillin–streptomycin–Fungizone (PSF; Cambrex, Walkersville, MD, USA) and culture medium. The culture medium contained 50:50 Dulbecco's modified Eagle's medium and F12 (Gibco, Carlsbad, CA, USA), 10% fetal bovine serum (FBS; Mediatech, Carlsbad, CA, USA), 1% non-essential amino acids (NEAA; Gibco, Carlsbad, CA, USA), 25 μg of L-ascorbic acid (Sigma, St Louis, Missouri,) and 1% PSF. The outer one-third of each meniscus was removed and the remainder was minced into small fragments (less than 1 mm^3^). Each minced meniscus was then placed in 30 ml of 2 mg/ml collagenase type II (Worthington Biochemical, Lakewood, NJ, USA) and transferred to an orbital shaker to be digested overnight at 37°C. After digestion, an equal volume of PBS was added to the mixture and centrifuged at 200 *g*. The bulk of the supernatant was removed, more PBS was added and the mixture was centrifuged again. This process was repeated until all the collagenase had been removed from the mixture, leaving behind a white pellet of meniscal cells. Cell counts from each meniscus were obtained with a hemocytometer. Cell viability was assessed with the use of a Trypan blue exclusion test and was found to be greater than 95%.

### Cell culture, passage and expansion

**Figure 1:**
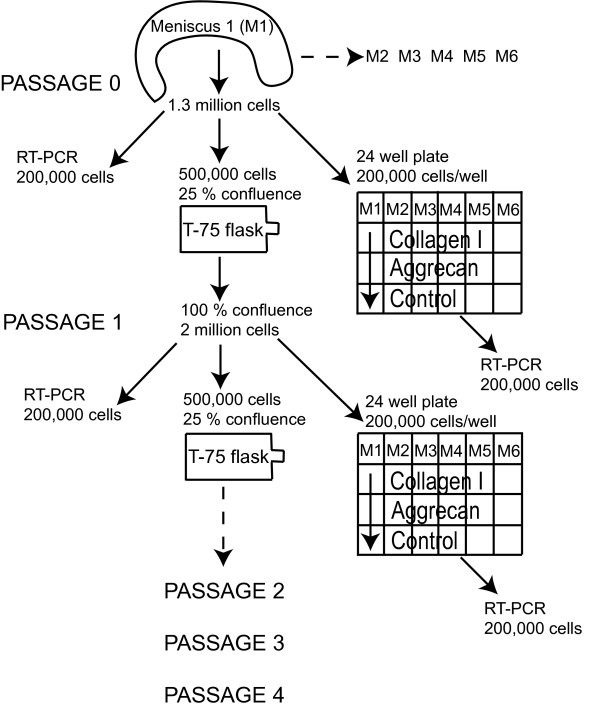
The overall experimental design. In brief, bovine meniscus cells were expanded through four passages in monolayer culture; 0.5 × 10^6 ^cells were expanded in a T-75 flask to confluence. At each passage time point, 0.2 × 10^6 ^cells were collected for RT-PCR, and 0.2 × 10^6 ^cells were plated on an aggrecan or collagen I two-dimensional surface coating or on a no coating control for 24 hours and then subsequently processed for RT-PCR. The gene expression profiles with passage and on the different protein coatings were then determined. *n *= 6 was used for all gene expression abundance evaluations.

From each meniscus, 1.3 × 10^6 ^cells were obtained, of which 0.2 × 10^6 ^were placed in 1 ml of TRIzol reagent (Invitrogen, Grand Island, NY, USA), 0.5 × 10^6 ^were plated on T-75 flasks at about 25% confluence, and the remaining 0.6 × 10^6 ^were divided into three equal groups and placed in a 24-well non-tissue-culture plastic plate coated with collagen 1 (Sigma, St Louis, Missouri, USA), aggrecan (Sigma, St Louis, Missouri, USA) or a non-protein control for 24 hours. Collagen I was dissolved in 0.1 M acetic acid and then diluted in water to a final concentration of 10 μg/cm^2 ^per 24-well plate. Aggrecan was soluble in water and was reconstituted to the same concentration. After plating, the 24-well plates were kept open in the cell culture hood and allowed to dry overnight. The cells were left to settle on the coatings for 1 day, and were then scraped off the bottom with a cell scraper and placed in 1 ml of TRIzol reagent. The cells in the T-75 flask were allowed to expand until 100% confluence and then passaged with trypsin/EDTA (Gibco, Carlsbad, CA, USA). The cells were counted with a hemocytometer and labeled as passage 1 (P1) cells. From this cell population, 0.2 × 10^6 ^cells were placed in 1 ml of TRIzol reagent, 0.5 × 10^6 ^were plated on T-75 flasks, and 0.6 × 10^6 ^were divided into three equal groups and placed in a 24-well non-tissue-culture plastic plate. This process was repeated until the fourth passage. The experimental design is shown in Figure 1.

### RNA isolation

Gene expression abundance of these cells was measured by means of quantitative real-time reverse transcriptase polymerase chain reaction (RT-PCR). In the first step, RNA was isolated from each sample that had previously been placed in TRIzol. Chloroform was added to each sample. The samples were then centrifuged at 12,000 r.p.m. for 15 minutes. Propan-2-ol was added to the supernatant and the sample was centrifuged again. The RNA precipitate was washed with 75% ethanol and then dissolved in diethyl pyrocarbonate (DEPC)-treated water. The concentration and purity of RNA was determined with a spectrophotometer (NanoDrop, Wilmington, DE, USA).

### Reverse transcriptase

After RNA isolation, the samples were normalized to 200 ng of RNA per sample, suspended in DEPC-treated water. Before reverse transcription, the RNA was treated with DNase to eliminate any DNA contamination in our samples. The RNA was then reverse transcribed to cDNA with a Stratascript™ First Strand Synthesis System (Stratagene, La Holla, CA, USA) in accordance with the manufacturer's protocol. In brief, random hexamers were added to each sample and the mixture was incubated at 65°C for 5 minutes, then cooled to 22°C for 10 minutes. Finally, to each sample 10× First strand buffer, RNase block, dNTPs and Stratascript enzyme were added. The samples were incubated at different temperatures starting at 25°C for 10 minutes, followed by 42°C for 60 minutes and finally 70°C for 15 minutes to terminate the reaction.

### Polymerase chain reaction

The cDNA obtained from the previous step was then amplified with a Rotor-gene 3000 real-time PCR machine (Corbett Research, Sydney, Australia). In brief, DEPC-treated water, 10× PCR buffer, MgCl_2_, dNTP, HotStar Taq and gene-specific primers and probes were added to the cDNA sample. The samples were heated to 95°C for 50 cycles, at 15 s per cycle, to denature and separate the strands of cDNA. The mix was then cooled to 60°C to allow the forward and reverse primers to anneal to the DNA strand and the HotStar Taq to elongate both primers in the direction of the target sequence.

Fluorescence measurements on the FAM, Cy5 and ROX channels were taken every cycle at 60°C to provide a quantitative, real-time analysis of the PCR reaction for specific genes. The genes of interest included collagen I, collagen II, aggrecan, COMP and glyceraldehyde-3-phosphate dehydrogenase (GAPDH). The forward and reverse primers and probe sequences for these genes are shown in Table 1. The primers and probes were optimized into triplexes such that (collagen I, COMP and GAPDH), and (collagen II, aggrecan and GAPDH) could be detected simultaneously.

**Table 1:** Primer and probe sequences of desired genes

Target gene (GenBank accession number, product size)	Forward primer (5'→3')	Reverse primer (5'→3')	Probe (5'→3')
Collagen-I (NM-174520, 97 bp)	CATTAGGGGTCACAATGGTC	TGGAGTTCCATTTTCACCAG	ATGGATTTGAAGGGACAGCCTTGG
Collagen-II^a ^(X02420, 76 bp)	AACGGTGGCTTCCACTTC	GCAGGAAGGTCATCTGGA	ATGACAACCTGGCTCCCAACACC
Aggrecan (U76615, 76 bp)	GCTACCCTGACCCTTCATC	AAGCTTTCTGGGATGTCCAC	TGACGCCATCTGCTACACAGGTGA
COMP (X74326, 72 bp)	TCAGAAGAGCAACGCAGAC	TCTTGGTCGCTGTCACAA	CAGAGGGATGTGGACCACGACTTC
GAPDH (U85042, 86 bp)	ACCCTCAAGATTGTCAGCAA	ACGATGCCAAAGTGGTCA	CCTCCTGCACCACCAACTGCTT

bp, base pairs; COMP, cartilage oligomeric matrix protein; GAPDH; glyceraldehyde-3-phosphate dehydrogenase. ^a^Collagen II primers detect both A and B isoforms.

### Gene expression efficiency and abundance

The efficiency of the PCR reactions was determined by taking dilutions of standard samples run in duplicate (1:1, 1:10, 1:100 and 1:1,000). The take-off cycle (*C*_*t*_) of the standard's slope was plotted against the logarithmic standards to determine the slope (*S*). The efficiency (*E*) was then determined with the following formula [23]:

*E *= 10^-1/*S *^

The abundance (*A*) of the gene was calculated by using the determined efficiency for the reaction, as well as the take-off cycle for the particular sample [24]:

*A *= (1 + *E*)^-*Ct *^

### Statistical analysis

Statistical analysis was performed with JMP IN™ software. A one-way analysis of variance (ANOVA) was run with five treatment groups (P0, P1, P2, P3 and P4), with passage number as a factor. To compare the effects of coating, a two-way ANOVA was run with coating and passage treated as factors. Coating had four treatment groups (collagen I coating, aggrecan coating, no coating control and no coating passage), whereas passage had five treatment groups (P0 to P4). If significance was observed with the ANOVAs, a post-hoc Tukey's Honestly Significant Difference test was run to pinpoint any specific differences among groups. The significant groups were further analyzed by crossing coating and passage factors to test for any specific differences observed between passages of different coating groups. *P *< 0.05 was considered significant for all statistical tests. All results are shown as mean ± SD.

## Results

### GAPDH as a verification gene

For clarity, the convention shown in Table 2 will be used hereafter. GAPDH expression was observed in more than 98% of the samples that were tested and was, thus, used as a verification gene. Samples with undetectable levels of GAPDH were not processed and were considered to be part of a failed reaction. No significant difference was observed in GAPDH expression between groups over passage.

**Table 2:** Terminology used to explain the different passage numbers as well surface coating groups

Passage	Explanation	Coating	Explanation
P0	Primary cells	Passage	Cells from P0 to P4 on T-75 flasks
P1	Cells that have undergone one passage	Collagen I coating	Cells from P0 to P4 on collagen I coating
P2	Cells that have undergone two passages	Aggrecan coating	Cells from P0 to P4 on aggrecan coating
P3	Cells that have undergone three passages	No coating	Cells from P0 to P4 on a water control
P4	Cells that have undergone four passages		

### Gene expression with passage

**Figure 2:**
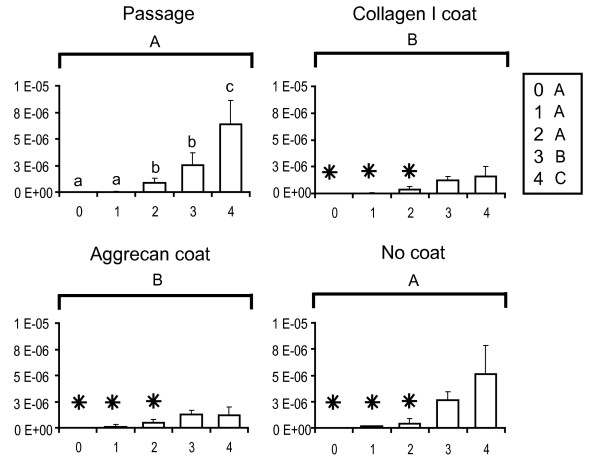
Collagen I gene expression profiles of meniscal fibrochondrocytes. The *x*-axis refers to the passage number, and the *y*-axis to the gene expression abundance (in the exponent notation used, 'E-*n*' stands for '× 10^-*n*^'). Small letters denote significant differences with passage, using a one-way analysis of variance (top left). Capital letters denote significant differences between levels (passage, collagen I coating, aggrecan coating and no coating), using a two-way analysis of variance. Stars denote groups that are not significantly different from values of the primary cells (that is, the P0 value in the top left panel), using an interaction term between the two factors.

The gene expression abundances for primary and passaged fibrochondrocytes are reported normalized to the amount of RNA per sample and are plotted for the genes of interest. These baseline passage values are shown in the upper left panels of Figures 2 (collagen I), 3 (collagen II), 4 (COMP) and 5 (aggrecan). Over four passages, a sharp 5,800-fold increase in gene expression was observed in collagen I levels (from (1.1 ± 1.2) × 10^-9 ^at P0 to (6.4 ± 2.5) × 10^-6 ^at P4), whereas a 70-fold decrease was observed with collagen II expression (from (1.2 ± 0.28) × 10^-8 ^at P0 to (1.8 ± 1.6) × 10^-10 ^at P4). COMP levels decreased sevenfold after the first passage (from (6.2 ± 4.6) × 10^-10 ^at P0 to (1.2 ± 1.2) × 10^-10 ^at P1) and then stayed relatively constant over the next three passages. Aggrecan abundance with passage did not seem to follow any particular trend. A fivefold decrease in gene expression was observed after the first passage (from (1.22 ± 0.417) × 10^-6 ^at P0 to (2.32 ± 1.20) × 10^-7 ^at P1). Gene expression was then upregulated in the second passage by about 25-fold (from (2.3 ± 1.20) × 10^-7 ^at P1 to (5.93 ± 2.45) × 10^-6 ^at P2) and then dipped again over the next few passages by about 1.5-fold (from (5.93 ± 2.45) × 10^-6 ^at P2 to (4.76 ± 2.17) × 10^-6 ^at P4).

**Figure 3:**
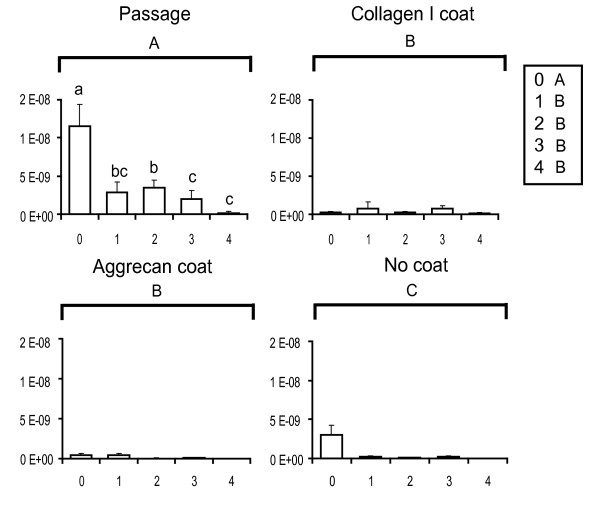
Collagen II gene expression profiles of meniscal fibrochondrocytes. The *x*-axis refers to the passage number, and the *y*-axis to the gene expression abundance (in the exponent notation used, 'E-*n*' stands for '× 10^-*n*^'). Small letters denote significant differences with passage, using a one-way analysis of variance (top left). Capital letters denote significant differences between levels (passage, collagen I coating, aggrecan coating and no coating), using a two-way analysis of variance.

**Figure 4:**
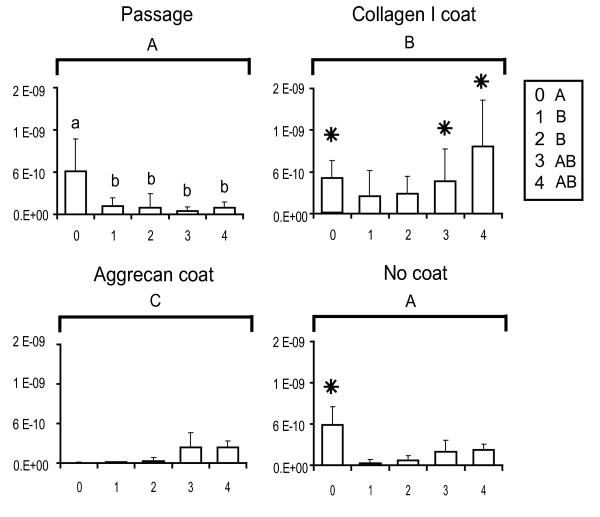
Cartilage oligomeric matrix protein gene expression profiles of meniscal fibrochondrocytes. The *x*-axis refers to the passage number, and the *y*-axis to the gene expression abundance (in the exponent notation used, 'E-*n*' stands for '× 10^-*n*^'). Small letters denote significant differences with passage, using a one-way analysis of variance (top left). Capital letters denote significant differences between levels (passage, collagen I coating, aggrecan coating and no coating), using a two-way analysis of variance. Stars denote groups that are not significantly different from values of the primary cells (that is, the P0 value in the top left panel), using an interaction term between the two factors.

**Figure 5:**
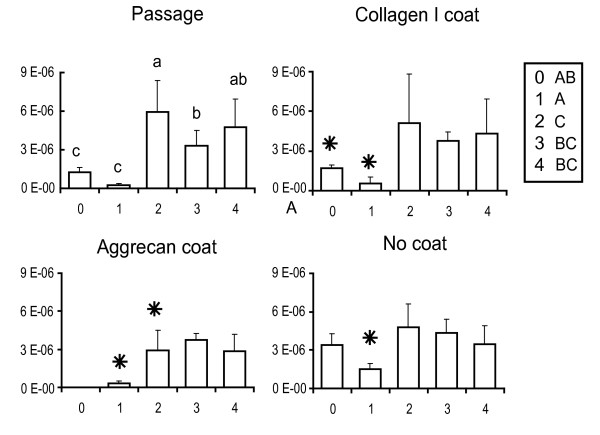
Aggrecan gene expression profiles of meniscal fibrochondrocytes. The *x*-axis refers to the passage number, and the *y*-axis to the gene expression abundance (in the exponent notation used, 'E-*n*' stands for '× 10^-*n*^'). Small letters denote significant differences with passage, using a one-way analysis of variance (top left). Capital letters denote significant differences between levels (passage, collagen I coating, aggrecan coating and no coating), using a two-way analysis of variance. Stars denote groups that are not significantly different from values of the primary cells (that is, the P0 value in the top left panel), using an interaction term between the two factors.

### Reversal attempts with protein coatings

Collagen I and aggrecan coatings were used to determine whether any changes in gene expression occurring as a result of monolayer passage could be reversed. The upper right and lower left panels of Figures 2 to 5 represent the reversal behavior of these protein coatings.

#### Collagen I

Cells placed on collagen I and aggrecan coatings showed significantly different gene expression profiles for collagen I over passage compared with the baseline passage and the no coating groups. Both protein coatings were found to decrease collagen I expression in the cells from the second to the fourth passage by 50% or more. In addition, the gene expression in the coating groups for all passages was within 20% of the P0 baseline abundance values.

#### Collagen II

Contrary to expectations, the decrease in collagen II expression observed over four passages was not reversed by either the collagen I or the aggrecan protein coating. In fact, both protein coatings induced a further downregulation of collagen II expression by about 50% or more at most passage time points. Interestingly, even the no coating control group showed a decrease in collagen II expression, as was observed with the protein coatings.

#### Cartilage oligomeric matrix protein

Significant differences were observed between the baseline passage group and the two coating groups. COMP expression in cells plated on collagen I protein coating was upregulated with each passage and had returned to baseline P0 levels by the third passage. In contrast, the aggrecan coating group showed some signs of reversal with passage; however, the effect was not as pronounced as in the collagen I coating group.

#### Aggrecan

None of the protein coating groups were found to have an effect on the expression of aggrecan in the passaged cells. Cells plated on the aggrecan protein coating tended to decrease aggrecan expression at all passages by at most twofold when compared with baseline values; however, the groups were not significantly different.

## Discussion

Cartilage tissue engineering studies generally require large numbers of cells that can be attained through expansion in monolayer. However, several experiments with articular chondrocytes and TMJ disc fibrochondrocytes have shown that phenotypic changes are common when dealing with passaged cartilaginous cells [17,18,25,26]. Further, gene expression reversal to baseline (P0) passage values after expansion has been met with minimal success [18,21]. Because similar studies have not been performed for meniscal fibrochondrocytes, in this study the degree of dedifferentiation and subsequent phenotype reversal via protein coatings were investigated by observing gene expression changes with passage. Significant differences in gene expression were observed over four passages for collagen I, collagen II and COMP, the first two being sensitive markers for the differentiation state of primary meniscal fibrochondrocytes [27]. In our gene expression reversal experiments, aggrecan and collagen I protein coatings aided in reversing collagen I and COMP expression to primary values; however, collagen II expression could not be reversed.

The morphology and phenotype of cartilaginous cells may be modulated by altering the culturing conditions. Meniscus cells cultured on alginate beads for 3 to 4 weeks were found both to resemble chondrocytes in morphology and to upregulate collagen II expression [27]. Similar results have been observed with dedifferentiated chondrocytes placed in three-dimensional hydrogels such as agarose or alginate [20,25]. In contrast, meniscus cells seeded for 1 day in monolayer seemed to be either rounded like chondrocytes or spindle-shaped like fibroblasts. However, after 1 week in monolayer, all cells spread and proliferated, exhibiting a morphology characteristic of fibroblasts [27]. It has been consistently shown in the literature that cartilaginous cells exhibiting a fibroblastic morphology express high levels of collagen I, with a downregulation in collagen II expression [18,26,28,29]. A similar result was observed in this experiment: expression of collagen I increased 5,800-fold over four passages, whereas collagen II expression decreased 70-fold. This observation may be attributed to dedifferentiation of meniscus cells in monolayer, in an analogous manner to dedifferentiation observed by Darling and Athanasiou [18]. However, the presence of multiple cell populations in the inner two-thirds of the meniscus that can proliferate at different rates must also considered as a potential contributor to the observed phenomenon. For instance, the rapid upregulation in collagen I expression, as normalized to total cells per sample, may be achieved by an increase in collagen I expression per cell, or, for multiple cell populations, an increase in the number of cells producing collagen I, or by a combination of these [7,26,27]. Similarly, the observed downregulation of collagen II may be a direct consequence of a decrease in the ratio of chondrocyte-like cells to fibroblast-like cells. Unfortunately, it is difficult to ascertain whether the passaged meniscus cells are composed of two cell populations or just one cell population expressing mainly fibroblastic genes. In future experiments examining gene expression it will be imperative to identify whether cell populations can be clearly distinguished before passage and, if so, to isolate the different cell types and analyze their proliferative, morphological and phenotypic properties separately to gain a better understanding of their individual contributions to the observed results.

Gene expression profiles of COMP, a pentameric glycoprotein found preferentially in the pericellular and territorial matrices of meniscus cells, were found to decrease significantly with passage [13,30]. Disruptions or mutations in the COMP structure have been linked with skeletal development disorders such as pseudoachondroplasia and multiple epiphyseal dysplasia, underlining the importance of COMP in the tissue [31,32]. A recent study with chondrocytes has shown that collagen II downregulation (the most common chondrocytic dedifferentiation marker) during monolayer passage is accompanied by a quicker downregulation of COMP [17]. Similar results were obtained in the present experiment, in which COMP expression decreased sevenfold after the first passage, although this was slower than the decrease in collagen II expression (15-fold after first passage). These results are in agreement with previous studies that have determined the function of COMP to be that of maintaining the integrity and properties of the collagen II network by bridging collagen II and collagen IX fibrils [17,33].

In addition to culturing conditions, the effect of aging on meniscus cells is a relevant topic of interest. Behavioral differences between immature and adult animals exist at the level of primary cells, and passaged adult cells may dedifferentiate to a different phenotype when compared with the cells examined in this study. Combining the results of this study with previous literature, such differences are expected to be small and the same trends are expected to hold. For instance, a protein expression study using skeletally mature and immature rabbit fibrochondrocytes expanded in primary and secondary monolayer culture showed no significant differences in sulfated proteoglycans and cell number [34]. With regard to the increased collagen I expression and decreased collagen II expression seen in that study as a result of passage, a more recent gene expression study by Hellio Le Graverand and colleagues showed that, in comparison with cells from immature tissue, adult primary cells expressed higher levels of collagen I and lower levels of collagen II [35]. This observation, taken together with past literature on the dedifferentiation of chondrocytes and the results of this study, indicates that adult cells are unlikely to be able to reverse this trend (that is, to begin to express more collagen II and less collagen I) [18]. The practical result of this study is therefore that, as with cells from immature tissue, with adult cells the already scarce collagen II expression is likely to be even lower with passage.

The rapid changes in gene expression of meniscus cells over passage are a matter of concern as this has important implications for future tissue engineering studies involving passaged meniscus cells. Several techniques have been used in the past to promote gene expression reversal of passaged chondrocytes and TMJ disc fibrochondrocytes back to primary cell values. These techniques have included the use of growth factors, three-dimensional hydrogels and protein coatings [18,20,22,25]. For meniscus cells, experiments have focused mainly on preventing dedifferentiation and stabilizing phenotype. For example, human meniscus cells cultured in alginate beads have been shown to obtain a round chondrocytic shape as well as to maintain the expression of collagen II over 3 to 4 weeks [27]. However, for most tissue engineering studies the cell population needs to be expanded. Culturing cells in three-dimensional environments, such as alginate, has been shown to promote protein synthesis while suppressing cell proliferation [18,29]. Unless an alternative medium that promotes both cell proliferation and phenotype retention is identified, gene expression reversal to primary cell values of expanded meniscus cells in a monolayer remains the most viable option.

We hypothesized that exposing passaged meniscus cells for 24 hours to collagen I or aggrecan, proteins abundantly present in the meniscus, would mimic the environment *in vivo *and be conducive to reversing lost phenotype. It is known that cells plated in monolayer interact with proteins present in FBS that are adsorbed on the cell culture flask [36,37]. This results in stimuli not generally encountered *in vivo*, prompting changes in cell morphology and surface marker expression [38]. An interesting result of the reversal study was that aggrecan coating decreased the expression of collagen I back to P0 baseline passage values. Previous studies in our laboratory have shown that dermal fibroblasts treated with insulin-like growth factor-I (IGF-I) and plated on an aggrecan surface coating adopted a chondrocytic phenotype and morphology, thus initiating the expression of collagen II with a downregulation of collagen I [39]. Passaged meniscus cells contain a high population of fibroblast-like cells; the observed decrease in collagen I expression was therefore not surprising [27]. However, the absence of IGF-I from the culture medium may have contributed to the lack of reversal of collagen II expression. It is plausible that IGF-I or other growth factors are essential for the expression of collagen II on fibroblast-like cells placed on an aggrecan protein coating [39]. However, the results of this study could also be a consequence of insufficient exposure time (namely 24 hours) to the aggrecan protein coating.

Collagen I protein coating was found to downregulate collagen I expression and upregulate COMP expression. The downregulation of collagen I expression may be attributed to a collagen I saturation effect experienced by the cells through integrins on the cell surface. It is known that cell-surface integrins can attach to region 1 (for example the I-domain of integrin α 2) of collagen I surfaces with a similar homology to the von Willebrand factor [40]. In addition, integrins also aid in the transmission of intracellular signals that can regulate cell growth, differentiation and motility [41]. It is therefore likely that similar integrins on passaged meniscus cells can sense the presence of excess collagen I in the vicinity and relay messages to the nucleus to downregulate collagen I expression. Proliferative rates of cells may affect gene expression as well, as is commonly observed in growth-plate chondrocytes [42]. It is has been shown that fibroblastic cells on three-dimensional collagen I matrices have lower proliferative rates than chondrocytic cells on the same surface, although the opposite is true in monolayer culture [43,44]. Because passaged meniscal cells exhibit mainly fibroblastic properties, the downregulation of collagen I may perhaps be attributed to the slower proliferation rate of these fibroblast-like cells. The upregulation of COMP gene expression back to primary fibrochondrocyte levels by the third passage was another exciting finding. COMP is an important marker for the dedifferentiation state of articular chondrocytes; its upregulation may therefore signal a resurgence of the chondrocytic population in the meniscus [17].

In this experiment, GADPH expression stayed relatively constant with passage and may be used to represent a housekeeping gene for future meniscus tissue engineering studies. GAPDH has often been employed as a useful housekeeping gene in RT-PCR studies not involving other standardization techniques. It is commonly believed that within the same tissue sample, GADPH mRNA expression levels are relatively constant, whereas they can vary considerably between tissue types [45]. Recent studies with fibrochondrocytes from the TMJ disc suggest that even though GADPH may be constant in different regions of the disc, there is a definite change in abundance with passage, a phenomenon not observed in passaged meniscal fibrochondrocytes [26].

## Conclusion

These data indicate that the cells of the inner two-thirds of the meniscus undergo significant changes during monolayer expansion and passage. They experience losses in major chondrocytic markers (collagen II and COMP) while experiencing gains in fibroblastic markers (collagen I). Reversal efforts to regain lost phenotype in passaged meniscus cells via protein coatings were successful for collagen I and COMP by means of collagen I and aggrecan coatings. However, reversal of collagen II gene expression proved to be unsuccessful. A lack of collagen II could result in structural breakdown of the tissue as well as preempt osteoarthritis [11,46,47]. It will therefore be important to investigate alternative vehicles for reversing losses in collagen II expression in passaged meniscus cells. These could include studying alternative protein coatings such as collagen II and decorin, adding growth factors such as transforming growth factor-β I (TGF-β I), fibroblast growth factor-II (FGF-II) and IGF-I to the culture medium, or culturing the cells in novel two-dimensional or three-dimensional environments that support proliferation while maintaining morphology [18,22,25,48-52].

## Abbreviations

ANOVA = analysis of variance; COMP = cartilage oligomeric matrix protein; DEPC = diethyl pyrocarbonate; ECM = extracellular matrix; FBS = fetal bovine serum; GAPDH = glyceraldehyde-3-phosphate dehydrogenase; IGF-I = insulin-like growth factor-I; PBS = phosphate-buffered saline; PSF = penicillin–streptomycin–Fungizone; RT-PCR = reverse transcriptase polymerase chain reaction; TMJ = temporomandibular joint.

## Competing interests

The authors declare that they have no competing interests.

## Authors' contributions

NJG and KAA conceived and designed the study. NJG performed all experiments, post-experimental assays, and statistical analyses described in the study, in addition to drafting the initial version of the manuscript. KAA supervised the study and oversaw the drafting of the manuscript. Both authors read and approved the final manuscript.
